# 

**Published:** 1918-12

**Authors:** 


### Subscription Acknowledgements

Remittances are hereby acknowledged, with thanks, from the following persons, for subscriptions to this Journal:

Dr. A. Aiken, Dr. D. T. Atkinson, Dr. C. A. Adams, Dr. W. M. Austin, Dr. J. B. Ainesworth (Starr, Miss.), Miss Eleanor Brackenridge, Dr. Frank L. Barnes, Dr. M. J. Bleim, Dr. R. P. Bishop, Dr. A. R. Bowman, Dr. J. F. Bell, Dr. M. M. Burnside, Dr. W. W. Brown, Dr. J. Q. Burton, Dr. R. C. Bristow, Dr. W. W. Cooper, John Creary Library, Dr. W. W. Callan, Dr. Edmond Doak, Dr. J. H. Florence, Dr. S. M. Gladney, Dr. F. C. Green, Dr. A. Garwood, Dr. Sophia Herzog-Huntington, Dr. J. W. Hicks, Dr. J. C. Holman, Dr. V. H. Hulen, (Cal.), Dr. L. Callender Johnson, Dr. E. A. Johnson, Dr. B. F. Kingsley, B. A. Kirkpatrick, B. E. Knolle, R. H. Knolle, John L. Lee, Dr. R. G. Loyd, Dr. R. R. Little, Dr. J. S. Lankford, Dr. R. R. LeMaster, Dr. R. C. Lacy, Dr. W. E. McCaleb, Dr. M. A. McBride, Drs. McReynolds and Seay, Dr. J. W. Macune, Dr. K. J. Mihills, Dr. John T. Moore, Dr. A. D. Nelson, Dr. J. A. T. Page, Dr. N. J. Pickett, Dr. John Preston, Dr. L. E. Parr, Dr. A. M. Phillips, (Miss.), Dr. J. H. Reuss, Dr. H. W. Robertson, Dr. Rodarte, Dr. W. B. Russ, Dr. T. B. Selman, Dr. D. L. Stephens, Dr. S. Sholars, Dr. G. M. Stephens, Dr. J. T. Spratt, Dr. John Turner, Dr. J. E. Thompson, Dr. W. R. Thompson, Dr. F. L. Thomas, Dr. G. W. Williams, Dr. G. W. Worthington, Dr. W. H. Walker, Dr. H. A. White, Dr. J. L. Womack, Dr. W. D. Yett, Dr. J. R. Wagner, Dr. F. S. White, Drs. Watt & Watt.

### Old Jokes That We Have Met.

"EVEN as death approaches," says a contemporary physician of national reputation, "the man of wit or the professional humorist is bound to have his little joke—even at his own expense.

"I was attending a well-known Western character who was dying by inches of our White Plague—consumption. In consequence the patient was much excited. To alleviate his sufferings one night, and to enable him to get a little sleep, I prescribed a hot mustard plaster to be applied to his chest.

"When the nurse approached with the poultice on a large diner plate, the poor fellow grinned and said with a dry chuckle, betwen two paroxysms of coughing:

"'Can't say that I like your bill of fare, doctor.'

"'Why not?' I inquired.

"'There's too much mustard for so little meat,' was the quick response."

---

"I THOUGHT you told me that your doctor had ordered you to quit drinking?" said Smith.

"Aw, these doctors don't know what they are talking about," replied Brown, as he stirred his highball. "I quit drinking for two days and I didn't feel a bit better."

---

DOCTOR (to operetta diva who wishes to be vaccinated):—"Shall I vaccinate your arm?"

DIVA:—"Heavens! No, of course not. Think of me as an artist with a scar on my arm! You must vaccinate me where it won't show."

DOCTOR:—"I think you had better take it internally."

---

AH, who would choose to be a Doctor—
A Microbe-stalking Pill-concoctor!
At 3 a. m. they ring his Bell
Because some Fellow's dined too well.
He has to leave a Joyous Frolic

Because a Baby gets a Colic;
And while subduing Mortal Ills
With Jalap, Ipecac and Squills,
He has to hear the Conversations
Of Patients, matching Operations;
And then, to crown his Pain and Strife;
They villify him here, in Life.

---

"OH, JOHN," *cried* the farmer's wife, "I'm afraid I've taken that dreadful disease!" "What makes you think so, dear?" he asked, alarmed, gathering the little frail woman into his arms and stroking the thinning hair, as she sobbed out the story of her fear upon his broad shoulder. "Well," she exclaimed, after I have gotten up, dressed myself and the children, cooked breakfast, washed the dishes, prepared the children for school, strained the new milk and set it away to cool, churned the butter, swept and dusted, done the ironing, given the baby his bath, cooked dinner and washed the dishes, sewed all afternoon, cooked supper and washed the dishes, undressed the children and put them to bed, and sat down for the evening, I am too tired to do any darning! I never used to feel so, it must be the hookworm."

---

A WESTERN Physician, who is quite aggressive, has the following notice displayed in his office:

"There i$ a little matter that $ome of my patient$ have $eemingly forgotten. It i$ unnece$$ary to $ay that I allude to the fee$ for my $ervic$. Money i$ needed in my bu$ine$$ and mu$t be had."

---

ZIMMERMANN, who was very eminent as a physician, went from Hanover to attend Frederick the Great in his last sickness. One day the king said to him: "You have, I presume, sir, helped many a man into another world?" This was rather a bitter pill for the doctor; but the dose he gave the king in return was a judicious mixture of truth and flattery. "Not so many as your majesty, nor with so much honor to myself."

